# Supplementary material for: Development and validation of an AI algorithm to generate realistic and meaningful counterfactuals for retinal imaging based on diffusion models
Source: PLOS Digit Health. 2025 May 15;4(5):e0000853. doi: 10.1371/journal.pdig.0000853 (PMC12080772; doi:10.1371/journal.pdig.0000853)
Supplement: S1 Text — (PDF) [file pdig.0000853.s001.pdf]

Generating Realistic and Meaningful Counterfactuals for Retinal Fundus and OCT Images using Diffusion Models

Supplementary Text

Ilanchezian, Boreiko et al.

Diffusion models

Forward diffusion is a Markov chain that gradually adds Gaussian noise to a starting image x_0:

$$q(x_t|x_{t-1}) = \mathcal{N}(x_t; \sqrt{1-\beta_t}x_{t-1}, \beta_t \mathbb{I}) \ , \tag{1}$$

where $t \in \{1, \dots, T\}$, β_t denotes a noise schedule such that $q(x_T|x_0) \approx \mathcal{N}(x_T; 0, \mathbb{I})$. Given x_0, the noisy images at any time step t can be also expressed in closed form:

$$x_t = \sqrt{\overline{\alpha}_t}x_0 + \sqrt{1-\overline{\alpha}_t}\epsilon \, , \epsilon \sim \mathcal{N}(0, \mathbb{I}), \tag{2}$$

where $\overline{\alpha}_t = \prod_{s=1}^{t}(1-\beta_s)$. Then, in the reverse diffusion process, the posterior $q(x_{t-1}|x_t, x_0)$, when conditioned on x_0, can be estimated using the Bayes Theorem [3]. The unconditioned posterior $q(x_{t-1}|x_t)$ is, however, intractable and has to be approximated by a parameterized distribution $p_\theta(x_{t-1}|x_t)$:

$$p_\theta(x_{t-1}|x_t) = \mathcal{N}(x_{t-1}; \mu_\theta(x_t, t), \Sigma_\theta(x_t, t)). \tag{3}$$

The mean and diagonal covariance of this distribution are predicted by DNNs denoted by $\mu_\theta(x_t, t)$ and $\Sigma_\theta(x_t, t)$, respectively. Briefly, these models are trained by optimizing a simplified loss function derived from the Variational Lower Bound (VLB) of the negative log likelihood $-\log p_\theta(x_0)$. The simplification involves learning the residual noise $\epsilon_\theta(x_t, t)$ at each time step and then expressing the mean $\mu_\theta(x_t, t)$ in terms of $\epsilon_\theta(x_t, t)$. $\Sigma_\theta(x_t, t)$ is modeled as an interpolation between β_t and $\tilde{\beta}_t = \frac{1-\overline{\alpha}_{t-1}}{1-\overline{\alpha}_t}\beta_t$ using a vector $\mathbf{v}$ that is output by the DNN. For further details about the loss functions and training, see [2]. Using $\mu_\theta(x_t, t)$ and $\Sigma_\theta(x_t, t)$, one can generate images from the data distribution $p(x)$ by starting with a sample from the standard normal distribution and iteratively reconstructing less noisy images at previous time steps from the current noisy image at time step t.

Adversarial attacks and robust training

An ℓ_p targeted adversarial attack for f_ϕ at x_0 produces a sample x, such that

$$\underset{c \in \{1, \dots, K\}}{\arg\max} \ f_\phi(x)_c = k, \quad x \in [0,1]^d \cap B_p(x_0, \varepsilon). \tag{4}$$

where $B_p(x_0, \varepsilon) := \{\hat{x} \in \mathbb{R}^d | \left\|x_0 - \hat{x}\right\|_p \leq \varepsilon\}$ is an ℓ_p ball around the original image x_0 with radius ε. One usually maximizes a surrogate loss L for this:

$$\underset{x \in [0,1]^d \cap B_p(x_0, \varepsilon)}{\arg\max} \quad L(f_\phi(x), k). \tag{5}$$

The loss function of the TRADES algorithm incorporates a term for the adversarial examples in addition to the standard cross-entropy loss:

$$\frac{1}{n}\sum_{i=1}^{n}\big[-\log\big(p_\phi(y_i|x_i)\big)+\beta\max_{x\in B_2(x_i,\varepsilon)}D_{KL}\big(p_\phi(\cdot|x)\,||\,p_\phi(\cdot|x_i)\big)\big], \tag{6}$$

where D_{KL} denotes the Kullback-Leibler divergence and β controls the trade-off between adversarial and plain training schemes. In our experiments, we set β to 6 [4, 5]. This process results in a classifier f_ψ which is robust to adversarial perturbations. Plain classifiers f_ϕ, on the other hand, are not robust to adversarial attacks and corresponds to training with only the cross-entropy loss i.e. β is 0.

Diffusion Visual Counterfactuals

In general, classifiers used in conjunction with diffusion models for conditional sampling are noise-aware, i.e., they are trained on noisy images which occur at various time steps in the diffusion process [2]. Consequently, the input to these classifiers includes the time step $t \in \{1, ..., T\}$ at which the image $x_t \in \mathbb{R}^d$ occurred. Since our classifiers are not noise-aware or time-dependent, that is to say the inputs are only the images $x \in \mathbb{R}^d$, we use an estimation of x_0 from a given noisy sample x_t as input to the classifier. We denote this estimated x_0 by $x_{0,dn}(x_t, t)$ and following Eqn. (2) it can be expressed as:

$$x_{0,dn}(x_t,t)\rightarrow\frac{x_t}{\sqrt{\bar{\alpha}_t}}-\frac{\sqrt{1-\bar{\alpha}_t}\epsilon_\theta(x_t,t)}{\sqrt{\bar{\alpha}_t}}, \tag{7}$$

where $\epsilon_\theta(x_t, t)$ can be calculated as a function of the mean $\mu_\theta(x_t, t)$ [2]. The reverse process transitions of a diffusion model guided by an external classifier which is not noise-aware are given by:

$$p_{\theta,\phi}(x_{t-1}|x_t,y)=Zp_\theta(x_{t-1}|x_t)p_\phi(y|x_{0,dn}(x_t,t)) \tag{8}$$

where Z is a normalization constant. Exact sampling from this distribution is intractable, however, it can be approximated by a Gaussian distribution in a way similar to the unconditional reverse transitions (Eqn. (3)) but with shifted mean [2] [1].

$$p_{\theta,\phi}(x_{t-1}|x_t,y)=\mathcal{N}(\mu_t,\Sigma_\theta(x_t,t)), \tag{9}$$

$$\mu_t=\mu_\theta(x_t,t)+\Sigma_\theta(x_t,t)\nabla_{x_t}\log p_\phi(y|x_{0,dn}(x_t,t)) \tag{10}$$

Along with distance regularization and adaptive parameterization, the final mean of the reverse transition probability is:

$$\mu_t=\mu_\theta(x_t,t)+\Sigma_\theta(x_t,t)\,\|\mu_\theta(x_t,t)\|_2\,\Gamma_{\text{DVC}} \tag{11}$$

$$\Gamma_{\text{DVC}}=\lambda_c\frac{\nabla_{x_t}\log p_\phi(y|x_{0,dn}(x_t,t))}{\|\nabla_{x_t}\log p_\phi(y|x_{0,dn}(x_t,t))\|_2}-\lambda_d\frac{\nabla_{x_t}d(x_0,x_{0,dn}(x_t,t))}{\|\nabla_{x_t}d(x_0,x_{0,dn}(x_t,t))\|_2} \tag{12}$$

When cone projection is used, the shift is given by:

$$\Gamma_{\text{DVC}}=\lambda_c\frac{\Gamma_{\text{cone}}}{\|\Gamma_{\text{cone}}\|_2}-\lambda_d\frac{\nabla_{x_t}d(x_0,x_{0,dn}(x_t,t))}{\|\nabla_{x_t}d(x_0,x_{0,dn}(x_t,t))\|_2}, \tag{13}$$

where

$$\Gamma_{\text{cone}}=P_{\text{cone}(\alpha,\nabla_{x_t}\log p_\phi(y|x_{0,dn}(x_t,t)))}\big[\nabla_{x_t}\log p_\psi(y|x_{0,dn}(x_t,t))\big], \tag{14}$$

λ_c and λ_d are positive constants and α is the angle of the cone, which we set to 30° following [1].

Sparse counterfactuals

For generating sparse counterfactuals, we used the log probability of the target class as a surrogate loss function (Eqn. (5)):

$$L(f_\psi(x), y) = -\log p_{f_\psi}(y|x) \tag{15}$$

References

1. Augustin M, Boreiko V, Croce F, Hein M. Diffusion Visual Counterfactual Explanations. In: Advances in Neural Information Processing Systems. vol. 35. Curran Associates, Inc.; 2022. p. 364–377.

2. Dhariwal P, Nichol A. Diffusion Models Beat GANs on Image Synthesis. In: Advances in Neural Information Processing Systems. vol. 34. Curran Associates, Inc.; 2021. p. 8780–8794.

3. Sohl-Dickstein J, Weiss E, Maheswaranathan N, Ganguli S. Deep Unsupervised Learning using Nonequilibrium Thermodynamics. In: Proceedings of the 32nd International Conference on Machine Learning. vol. 37 of Proceedings of Machine Learning Research. PMLR; 2015. p. 2256–2265.

4. Zhang H, Yu Y, Jiao J, Xing EP, Ghaoui LE, Jordan MI. Theoretically Principled Trade-off between Robustness and Accuracy. In: ICML; 2019.

5. Stutz D, Hein M, Schiele B. Relating Adversarially Robust Generalization to Flat Minima. In: ICCV; 2021.
