# Supplementary material for: Development and validation of an AI algorithm to generate realistic and meaningful counterfactuals for retinal imaging based on diffusion models
Source: PLOS Digit Health. 2025 May 15;4(5):e0000853. doi: 10.1371/journal.pdig.0000853 (PMC12080772; doi:10.1371/journal.pdig.0000853)
Supplement: S1 Table — Top 2 rows shows evaluation of plain binary classifiers trained with and without including the mild class. The rows below corresponding to Robust shows the evaluation of robust binary classifiers that are trained with different ϵ values and initializations - ImageNet indicates that model is finetuned from ImageNet pre-trained plain model and Madry Robust indicates that model is finetuned from an adversarially robust model trained on the ImageNet dataset. Bottom 3 rows shows performance of 5-class models using different ϵ values. (PDF) [file pdig.0000853.s002.pdf]

Generating Realistic and Meaningful Counterfactuals for Retinal Fundus and OCT Images using Diffusion Models

Supplementary Text

Ilanchezian, Boreiko et al.

Model	ϵ	Pre-training	Mild class excluded	**Binary Classification**		**5-Class Classification**	
				acc.	bal acc.	5-class acc.	Quad. κ
Plain	-	ImageNet	No	92.39	80.67	-	-
	-	ImageNet	Yes	94.68	79.74	-	-
Robust	0.1	ImageNet	No	90.75	60.94	-	-
	0.05	ImageNet	No	90.65	65.78	-	-
	0.1	Madry Robust	No	90.49	62.59	-	-
	0.05	Madry Robust	No	90.18	66.86	-	-
	0.01	Madry Robust	No	90.03	74.35	-	-
	0.1	Madry Robust	Yes	89.68	63.32	-	-
	0.05	Madry Robust	Yes	90.39	68.04	-	-
	0.01	Madry Robust	Yes	90.95	76.72	-	-
Robust (5-class)	0.03	ImageNet	No	-	-	83.41	0.41
	0.02	ImageNet	No	-	-	83.80	0.45
	0.01	ImageNet	No	-	-	83.69	0.51

Table 1. Performance of plain and robust fundus binary and 5-class classifiers under different settings. Top 2 rows shows evaluation of plain binary classifiers trained with and without including the mild class. The rows below corresponding to Robust shows the evaluation of robust binary classifiers that are trained with different ϵ values and initializations - ImageNet indicates that model is finetuned from ImageNet pre-trained plain model and Madry Robust indicates that model is finetuned from an adversarially robust model trained on the ImageNet dataset. Bottom 3 rows shows performance of 5-class models using different ϵ values.
