# Supplementary figures and images for: Development and validation of an AI algorithm to generate realistic and meaningful counterfactuals for retinal imaging based on diffusion models
Source: PLOS Digit Health. 2025 May 15;4(5):e0000853. doi: 10.1371/journal.pdig.0000853 (PMC12080772; doi:10.1371/journal.pdig.0000853)

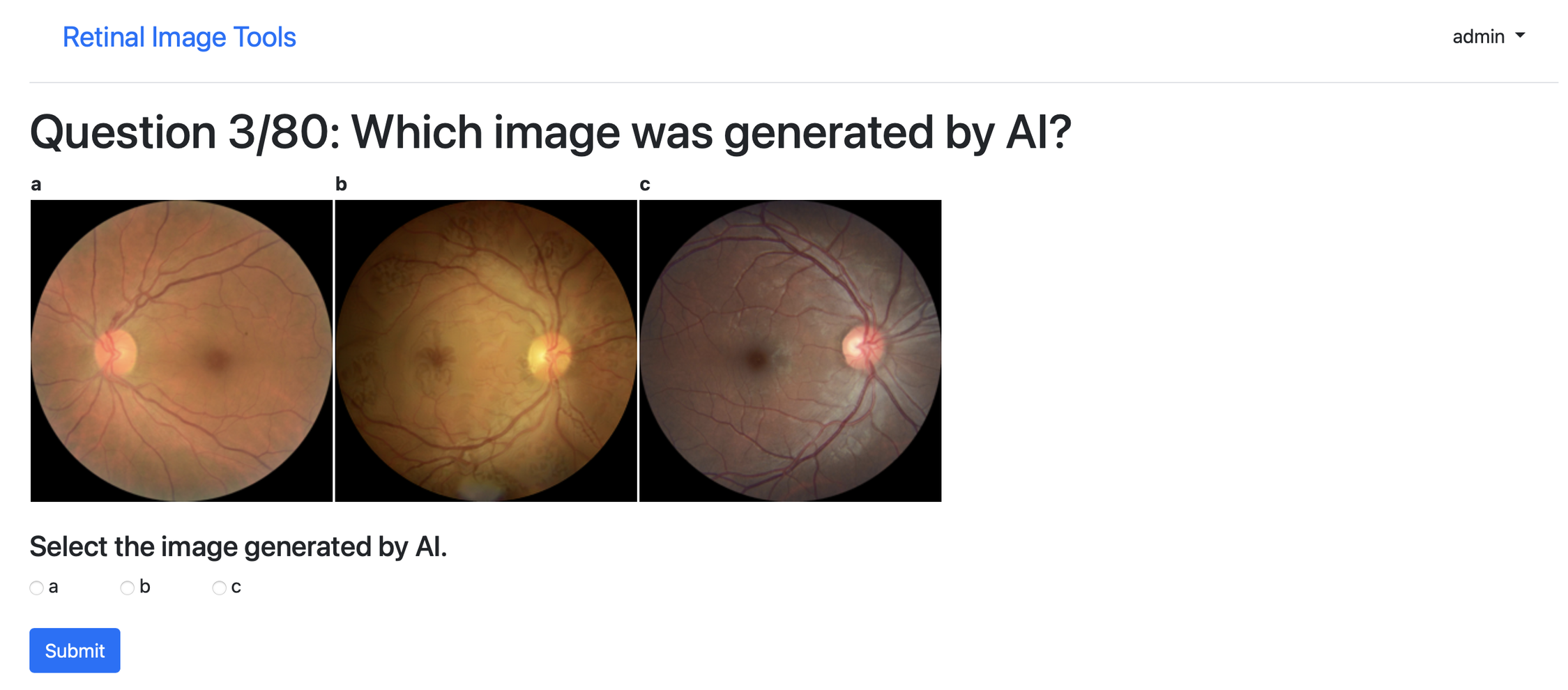

Supplement: S1 Fig — Three images are shown on the page where two are real and one is generated. User is asked to select the generated image. (TIF) [file pdig.0000853.s003.tif]

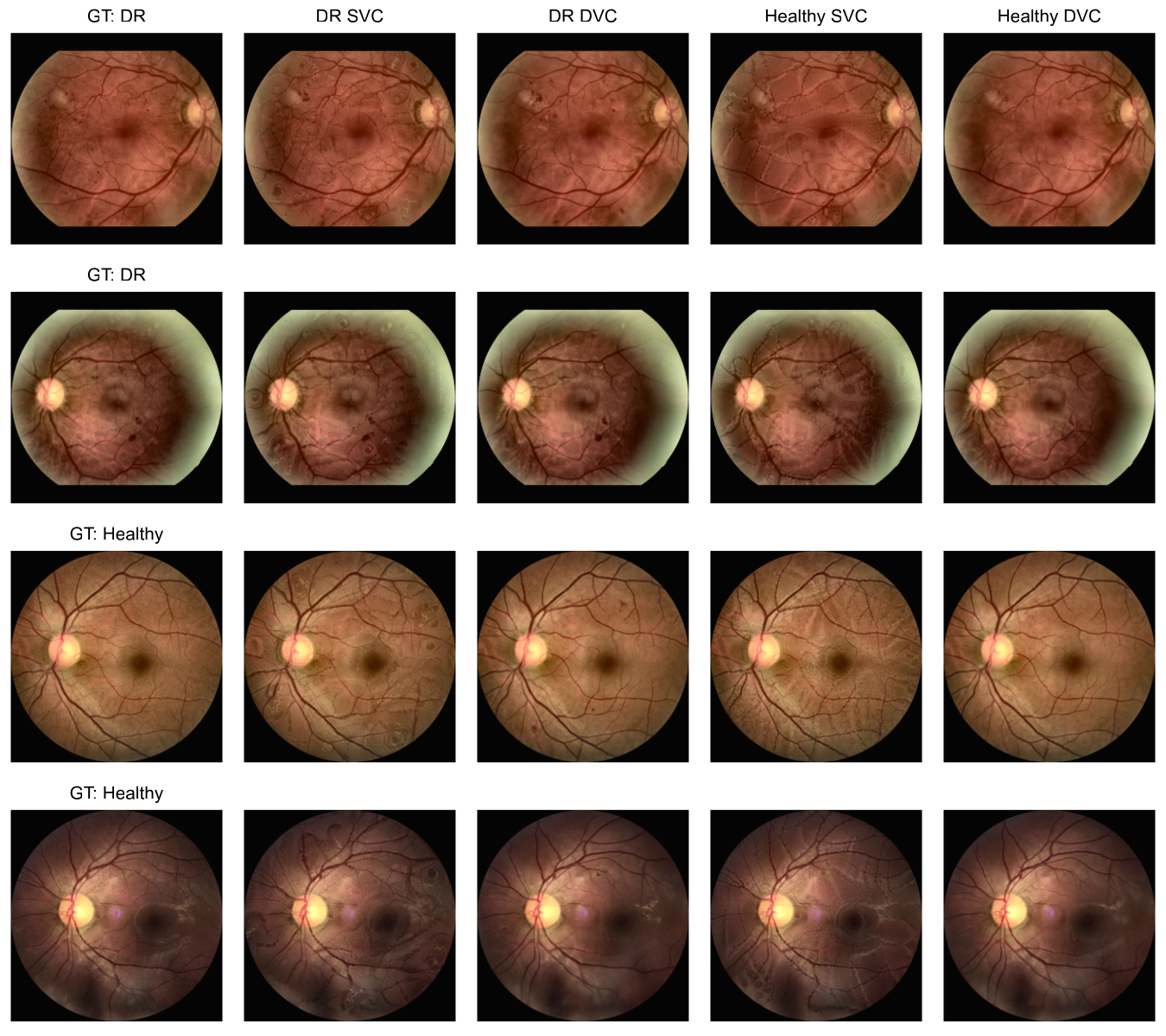

Supplement: S2 Fig — Top two rows show counterfactuals from DR fundus images. Bottom two rows show counterfactuals from healthy images. In all cases, changes in DVCs are more realistic compared to SVCs. (TIF) [file pdig.0000853.s004.tif]

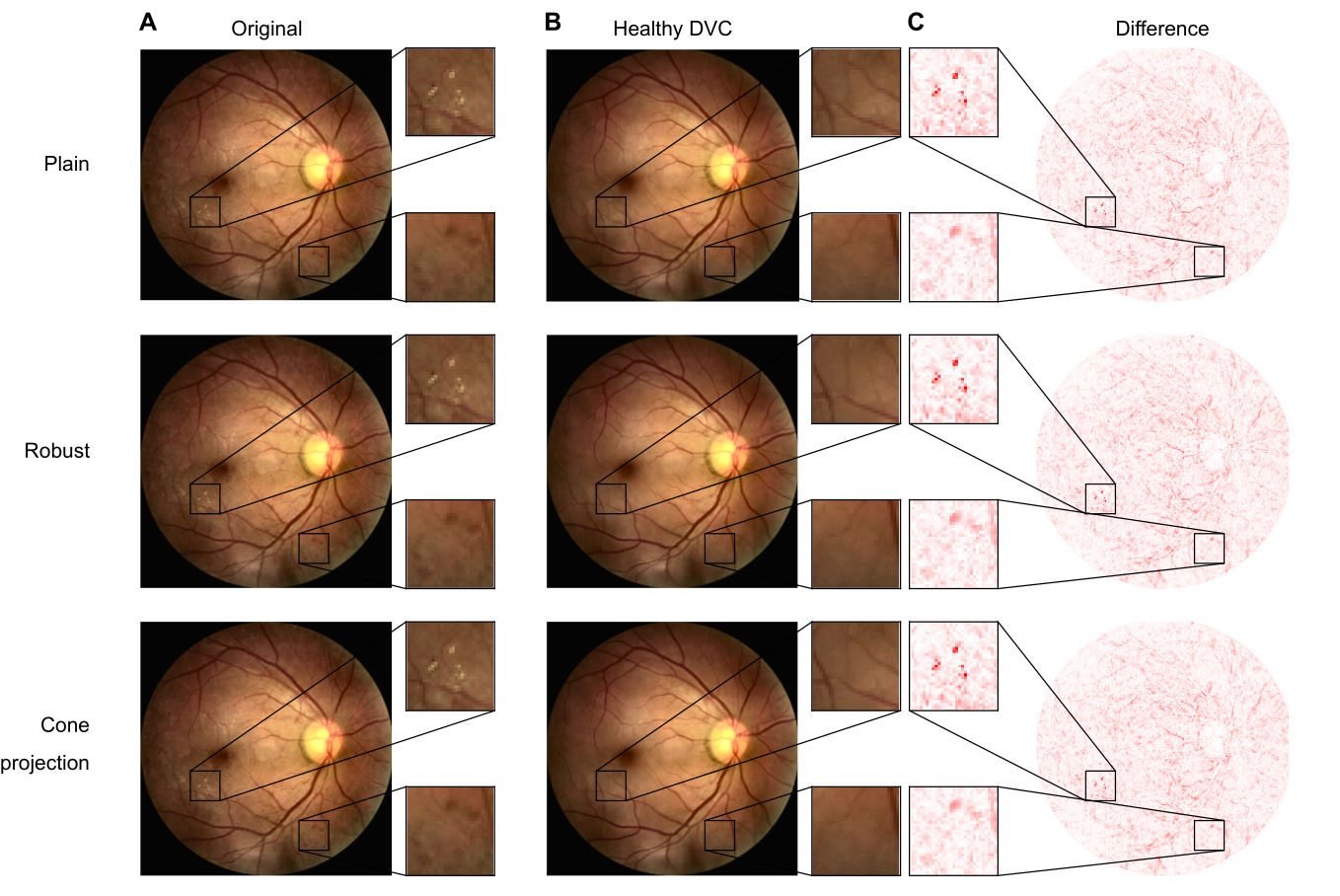

Supplement: S3 Fig — Original image with ground truth label DR. b. DVC to the healthy class. Plain model (top row) removes lesions to a similar extent as robust (middle row) and cone projection (bottom row). DVCs to healthy class are more easily generated than to DR class. c Difference maps between the original DR image and generated healthy counterfactual highlighting lesion locations. (TIF) [file pdig.0000853.s005.tif]

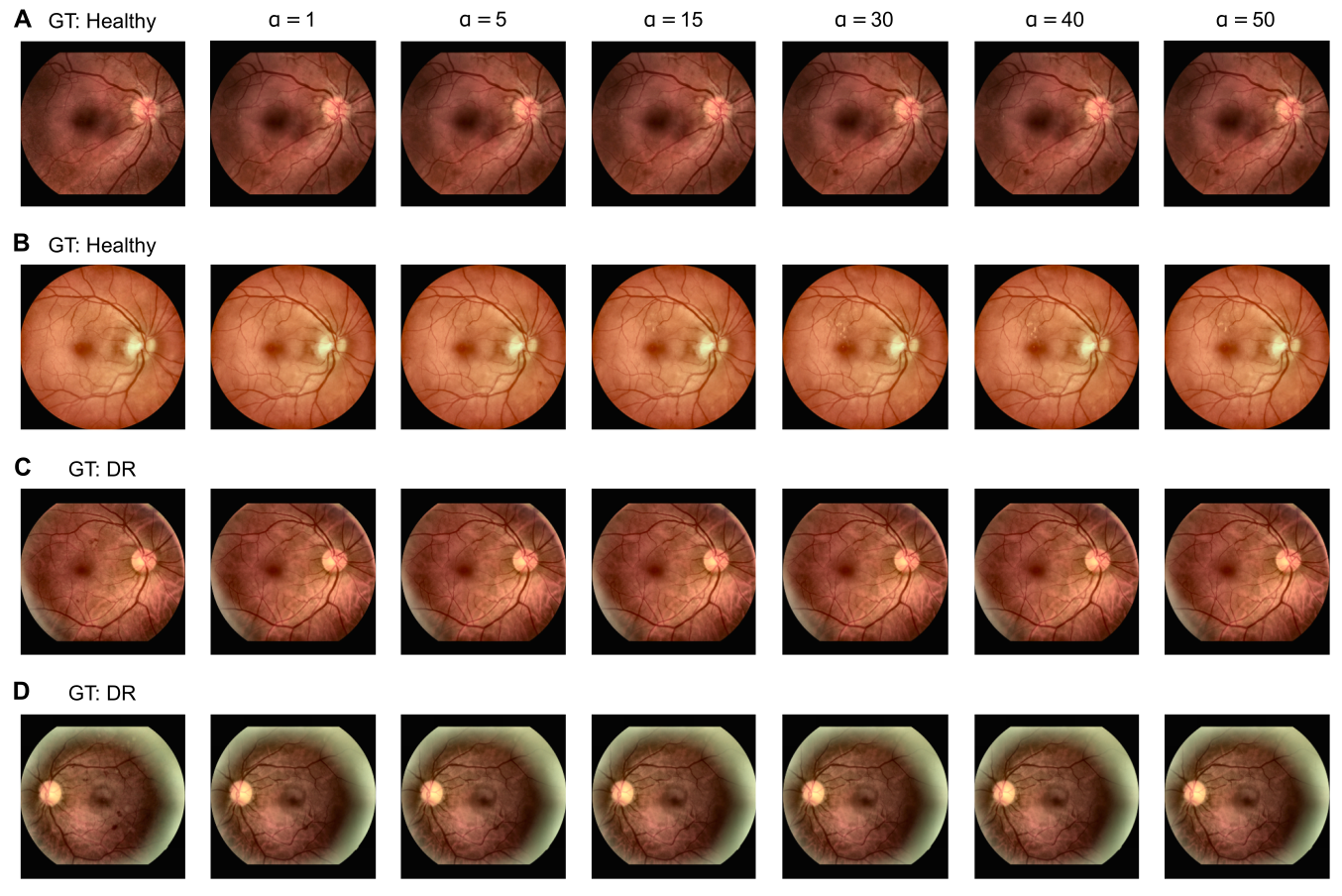

Supplement: S4 Fig — DR DVCs of healthy fundus images with different values of α∈{1,5,15,30,40,50}. Lower angles correspond to a larger weight of plain classifier gradients. With low values α∈{1,5,15} disease signs are not seen on the DVCs. On the other hand, setting α higher than 30 results in the introduction of meaningful lesions on the originally healthy images implying that the robust classifier’s gradients play a significant role in generating DR DVCs. c–d Healthy DVCs of DR fundus images with same values of α as in a–b. In this case, lesions are effectively removed to generate healthy DVCs even with a lower angle. Plain classifier alone is able to generate a healthy DVC effectively from an originally DR image. This is in line with our findings derived from Fig 5 and S3 Fig and summarized in Sect Realistic counterfactual examples require robust classifiers. (TIF) [file pdig.0000853.s006.tif]

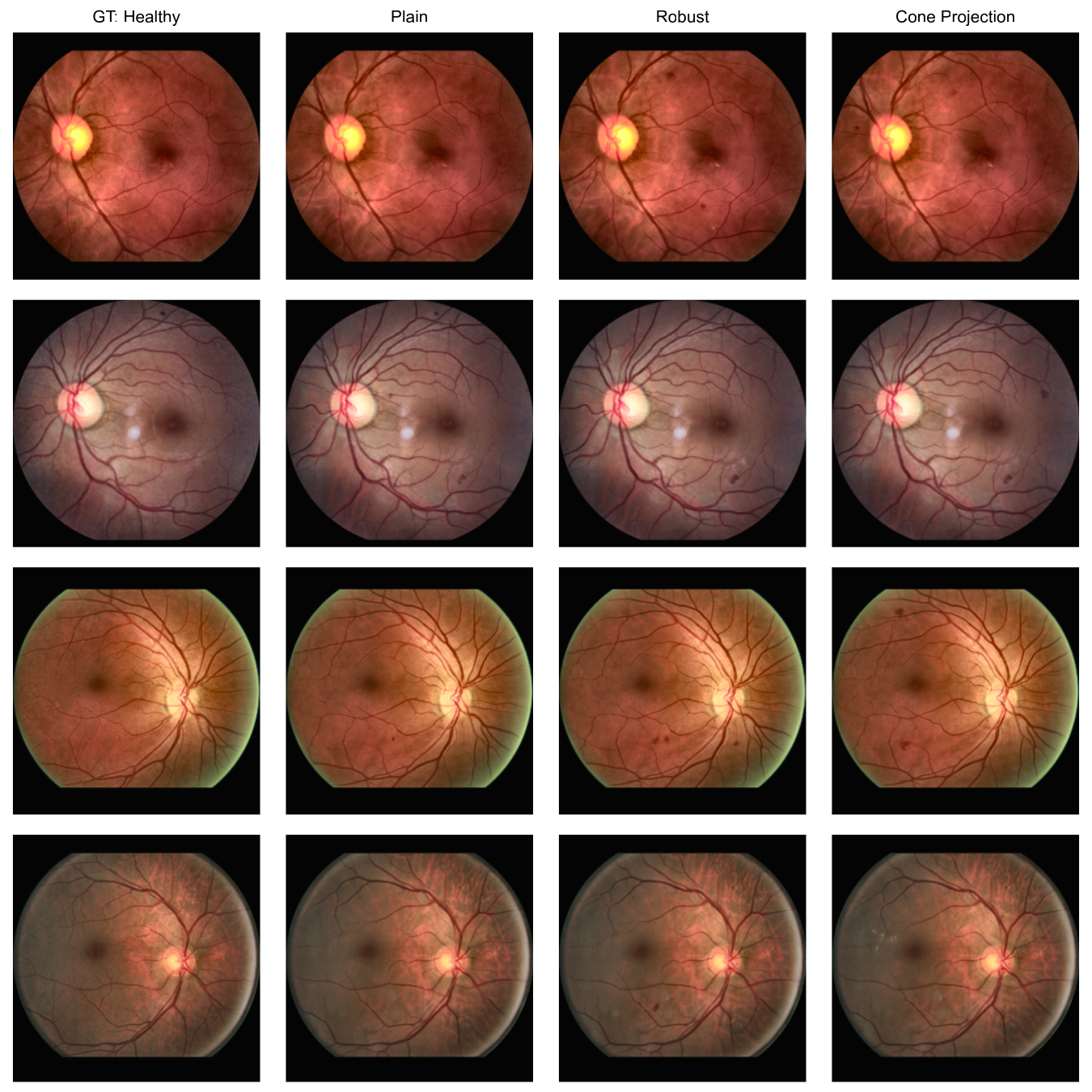

Supplement: S5 Fig — In all examples, plain models either show no or fewer and weaker lesions compared to robust and cone projection models. (TIF) [file pdig.0000853.s007.tif]

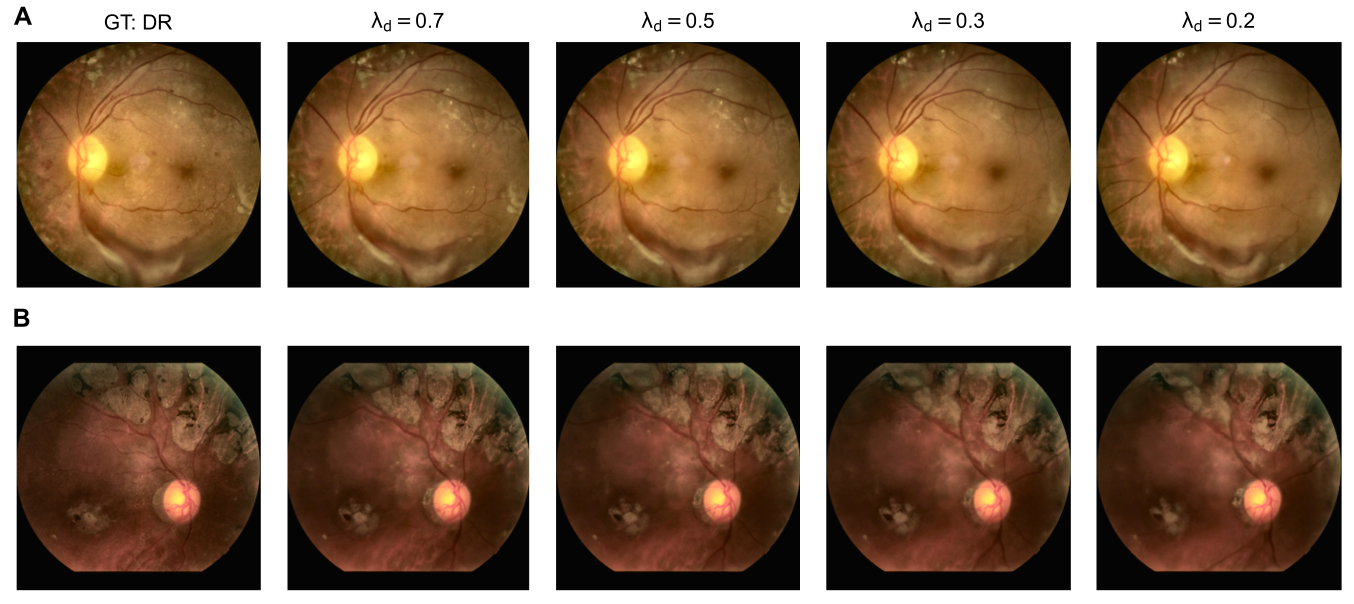

Supplement: S6 Fig — In such extreme cases, even a small regularization of 0.2 is not sufficient to convert the image to healthy. (TIF) [file pdig.0000853.s008.tif]

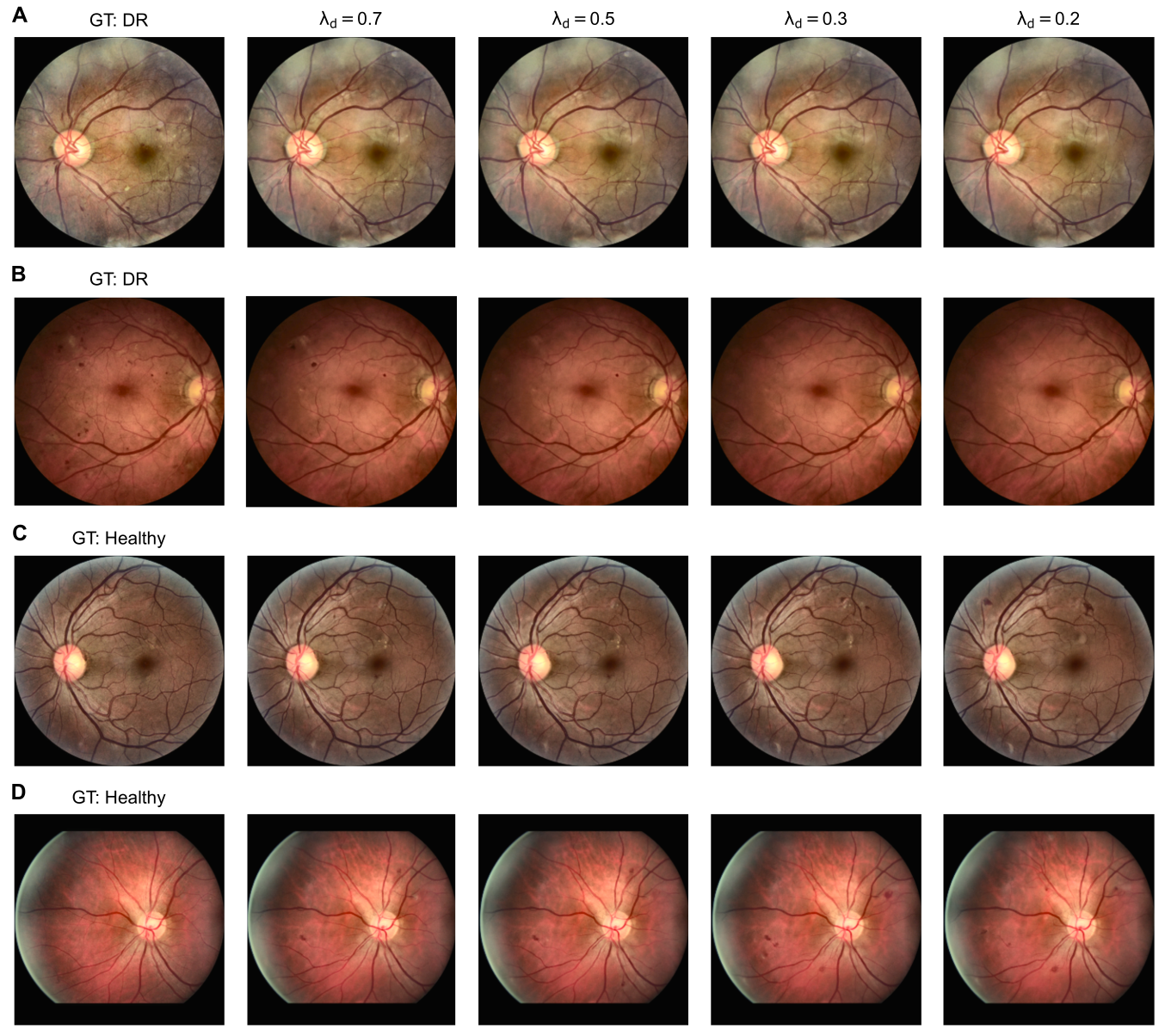

Supplement: S7 Fig — Healthy DVCs of DR fundus images with different values of λd. With λd=0.5, examples are converted to the healthy class with either no lesions ( a) or very few remaining lesions ( b) such as in the mild DR class. c–d DR DVCs of healthy fundus images with varying λd. Here too, with λd=0.5, the DVC adds enough lesions to change the decision of the classifier to the DR class with high confidence. (TIF) [file pdig.0000853.s009.tif]

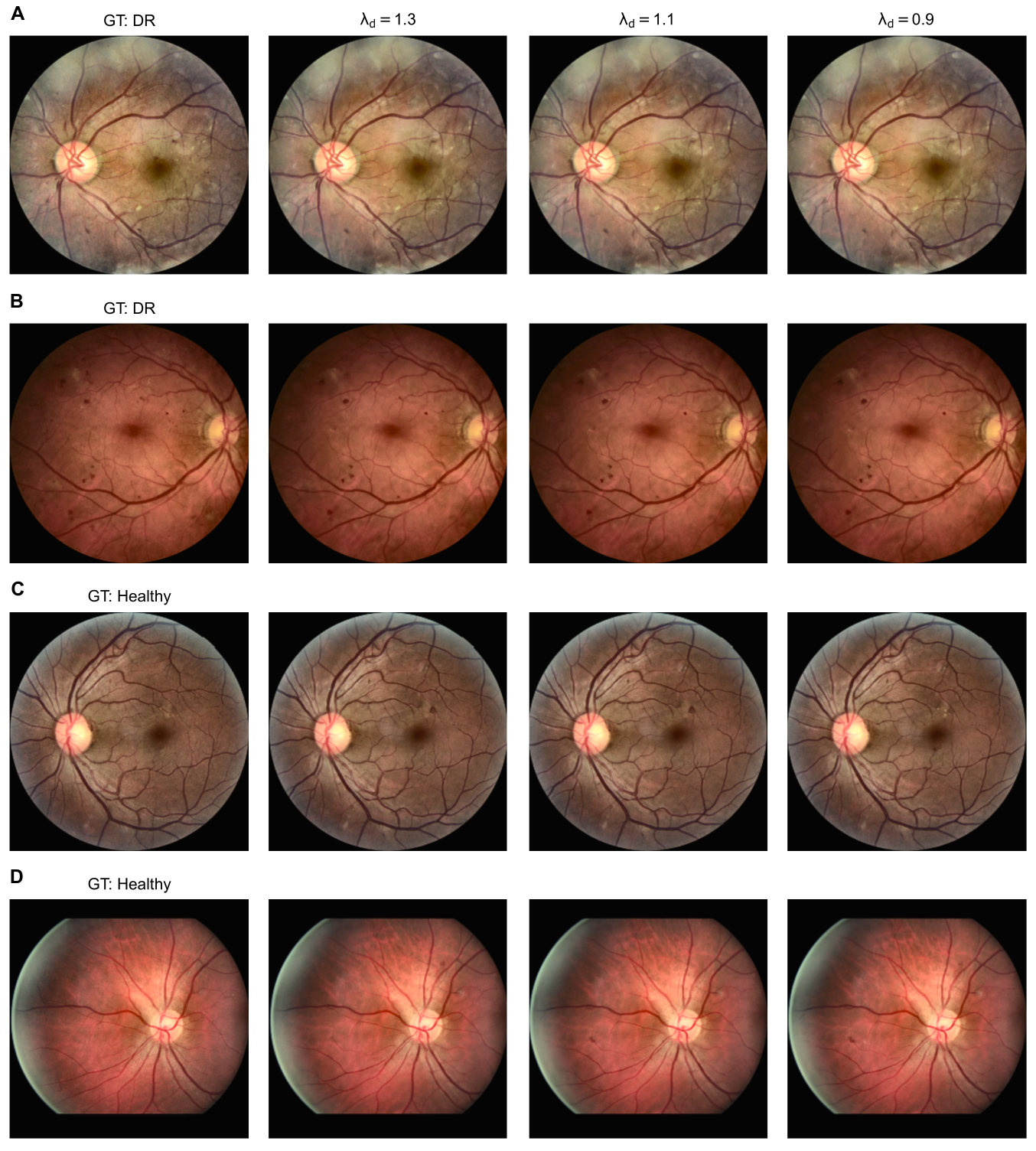

Supplement: S8 Fig — Healthy DVCs of DR fundus images with different values of λd∈{0.9,1.1,1.3} . With such high λd values, DR lesions are not removed and the resulting DVCs do not appear to be healthy c–d DR DVCs of healthy fundus images with same higher values of λd as in a–b. Here too, the lesions added are too subtle and hard to notice, thus achieving a low confidence for the DR class. (TIF) [file pdig.0000853.s010.tif]

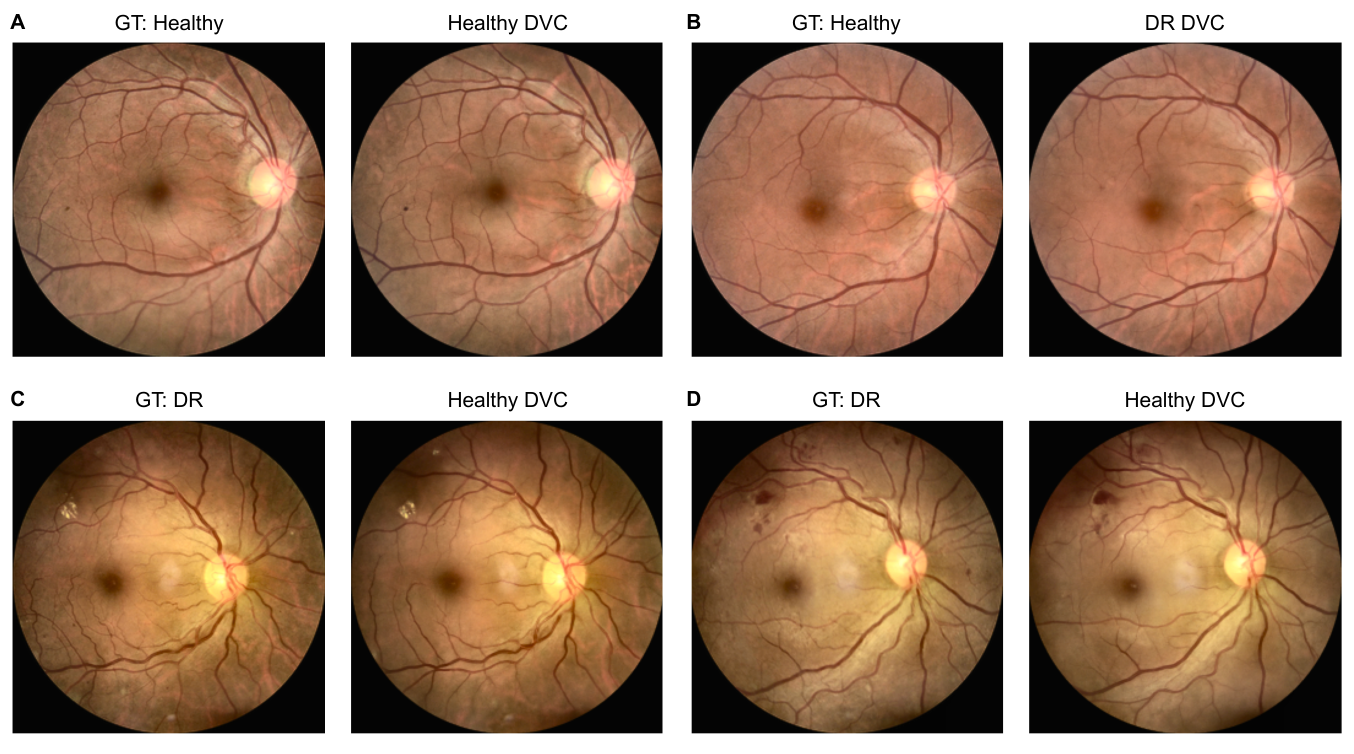

Supplement: S9 Fig — DVC of a healthy image to the healthy class only brightens an artifact present in the image but is still predicted as healthy with high confidence. b. DR DVC of a healthy example does not exhibit any disease related lesions. c–d. Healthy DVCs of DR examples still show disease related lesions. In c, one of lesions appears brighter in the DVC. (TIF) [file pdig.0000853.s011.tif]

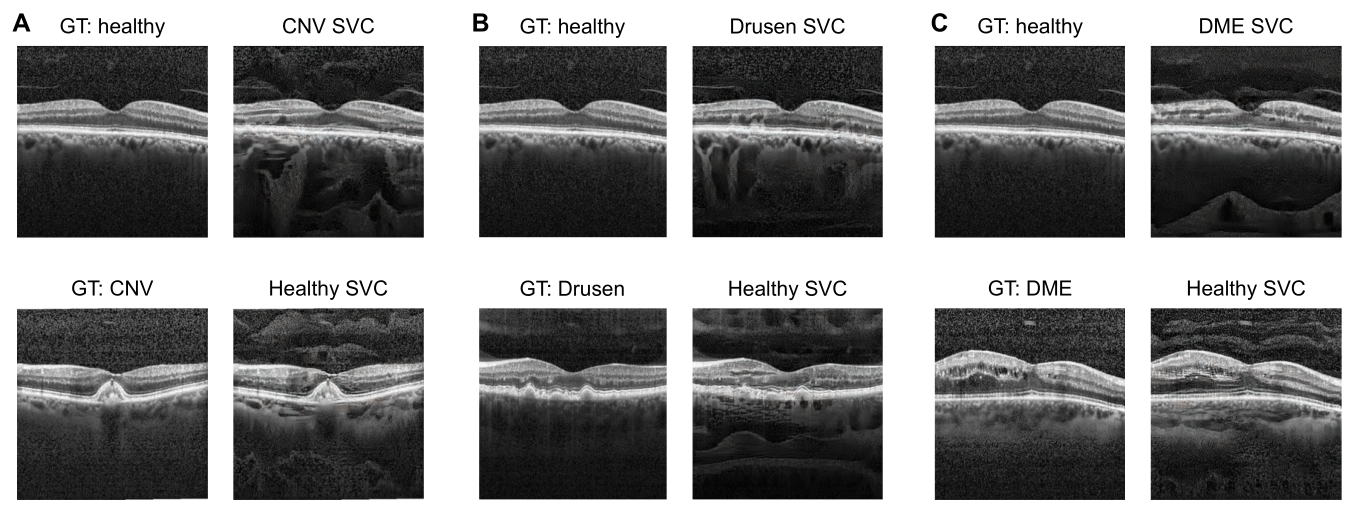

Supplement: S10 Fig — SVC from healthy to CNV (top) and from CNV to healthy (bottom). b–c. Same as a for classes drusen ( b) and DME ( c). While SVCs to the diseased classes introduce the respective disease signs, they also produce several artifacts on the background. On the other hand, the method struggles to convert the diseased classes back to healthy especially when the signs of disease are extreme as in the CNV case ( a). For drusen and DME the SVC attempts to cover the disease signs although in an unrealistic manner. (TIF) [file pdig.0000853.s012.tif]
