# Supplementary material for: Development and validation of an AI algorithm to generate realistic and meaningful counterfactuals for retinal imaging based on diffusion models
Source: PLOS Digit Health. 2025 May 15;4(5):e0000853. doi: 10.1371/journal.pdig.0000853 (PMC12080772; doi:10.1371/journal.pdig.0000853)
Supplement: Attachment — Submitted filename: PloS DH Rebuttal.pdf [file pdig.0000853.s014.pdf]

Reviewer #1: This manuscript presents a novel approach to generate realistic and clinically meaningful retinal fundus images using diffusion models combined with classifier guidance, bridging advanced computer vision methods with domain-specific studies in medical imaging. The authors demonstrate that their model can generate high quality counterfactuals for both CFP and OCT images, validated by technical and clinical experts. The work is technically sound, with a clear explanation of the methods, open-source code and dataset sources, and rigorous evaluation results.

The main contribution of this work is the technical innovation of computer vision methods (generating counterfactuals using diffusion models guided by classifiers) in the clinical domain. The combination of diffusion models, adversarially robust classifiers and cone projection is well motivated and effectively implemented. What's more, expert evaluation by AI specialists and ophthalmologists lends credibility to the results. The three-way odd-one-out task and clinical relevance assessment provide strong evidence that the generated images are realistic and clinically meaningful.

The authors also evaluate the technical improvements over the baseline and analyse the effect of regularisation strength on the generated images. Supplementary materials with additional visual examples and mathematical details increase the transparency and reproducibility of the work.

The manuscript is well written and logically structured, with clear explanations of methods and results. The use of many visual examples throughout the manuscript helps to effectively illustrate the key points.

We thank the reviewer for the concise summary and for appreciating our contributions.

However, there are some areas for improvement. Firstly, while the authors discuss the role of robust classifiers in generating high quality counterfactuals, a more systematic and quantified ablation study comparing different classifiers or classifier settings could improve technical soundness and provide deeper insights into the behaviour of AI models in clinical terms.

This is an excellent point – indeed, if the focus of our paper had been on explaining classifiers like in Boreiko et al. (2022, MICCAI), it would have been natural to ask if the counterfactuals depend on the classifier and the classifier settings used. To some extent this is explored in Fig. 5, where we compare a plain and a robust classifier as well as their combination.

However, as our focus is on validating a particular counterfactual generation method with human experts and we already have 9 main figures and 10 supplementary figures, many of which perform extensive ablation experiments regarding various parts of the algorithm, we defer an in-depth analysis of the effects of different classifiers to future work. Yet, we have extended the Discussion mentioning the potential dependence of counterfactuals on the classifier and its settings and we have added a Supplementary Table showcasing classification in different classifier settings.

Another area for improvement is that, although the author acknowledges that the imbalance of DR datasets affects the quality of the generated counterfactuals for underrepresented classes (severe and proliferative diabetic retinopathy), reporting the statistical results for each class will provide a more nuanced understanding of the model's performance and help identify the specialised classes for improvement. Furthermore, as this work aims to generate synthetic

retinal images, exploring potential solutions such as data argumentation could deepen the contribution of this study in addressing real-world dataset imbalances.

We refer the reviewer to section 3.4 where we state that diffusion counterfactuals to severe and proliferative DR did not work with target probabilities below 0.25, likely due to an underrepresentation of such images in the data (they make up only about 1% of the original training data). We attempted to mitigate this shortcoming with additional datasets and extensive oversampling/data augmentation to balance classes, which was unfortunately still not successful. We now mention this more prominently in the Discussion. We note however, that our OCT example shows that given a suitably balanced dataset, our algorithm is in principle able to also model larger structural changes.

Also, although the authors provide many visual examples of generated images compared to real images, the introduction of more detailed textual explanations, especially for added or detected visual features in counterfactuals, would enhance the clinical relevance of the work. An audience without a deep background in ophthalmology will benefit from this improvement.

We thank the reviewer for pointing this out. We have added more explanations to the text to increase accessibility.

Overall, this manuscript makes a valuable contribution to AI medical image synthesis. The use of diffusion models that combine classifiers to generate clinically meaningful realistic counterfactuals would benefit not only retinal fundus images, but also other medical images. Expert evaluation added credibility to the results. With further analysis and clinical insight, this work has the potential to impact medical imaging and medical education, as well as address real-world challenges such as data set imbalance.

Thank you again for your positive assessment!

Reviewer #2: Uploaded in word document.

Summary: This is a really nice manuscript that presents a novel AI-based approach to generating realistic and meaningful counterfactual retinal images using diffusion models guided by disease classifiers. The authors propose a method that can illustrate hypothetical scenarios, such as how a healthy fundus image would appear if the subject had diabetic retinopathy (DR). The study validates the approach through a combination of classifier-guided diffusion models and user studies involving ophthalmologists and AI experts. The results and methods here are very useful to the field and have potential for future clinical use, yet require a few more points to be considered before publication.

We thank the reviewer for their positive evaluation.

Major comments:

• Could some of the diseased counterfactuals be generated by changing the blood vessel morphology (e.g., blood vessel density, tortuosity, etc.) in the image rather than just creating lesions? This could potentially explain why you might be seeing barely noticeable lesions in some of the DR DVCs, like discussed in figure 5. Additionally, if these techniques are eventually to be used to help train opthalmologists to diagnose disease, and if DR is diagnosed by means other than just lesions, then it would be good to make sure the images being generated for counterfactual purposes reflect all the ways that DR may be diagnosed. Therefore, I think it

would be useful to also test the morphological vessel changes that occur to a disease/healthy generated image. This article (https://www.nature.com/articles/s41467-024-52334-1) seems to show that there are quite a few morphological changes that are coincident with DR.

We thank the reviewer for bringing this up. The DR classification scale proposed by Wilkinson et al. (2003) indeed see prominent venous beading in more than two quadrants as a diagnostic criterion for severe DR. As our model did not capture the changes due to severe and proliferative DR well and generating counterfactuals for these classes did not work (see above), we could not test this idea specifically. However, Fig. 6 (bottom) works with an example that shows some vascular changes due to DR, e.g. in the blood vessels extending to the right from the optic disc. In the healthy counterfactuals, these blood vessels look straighter and less like beads, providing qualitative evidence that our model indeed captures also some vascular characteristics. We added a description of this observation to the text and mention it also in the Discussion.

Wilkinson, C. P., Ferris III, F. L., Klein, R. E., Lee, P. P., Agardh, C. D., Davis, M., ... & Global Diabetic Retinopathy Project Group. (2003). Proposed international clinical diabetic retinopathy and diabetic macular edema disease severity scales. *Ophthalmology*, *110*(9), 1677-1682.

• The authors indicate that they used publicly available datasets (available online) but the link they shared for eyepacs data only sends you to the eyepacs news announcements part of the eyepacs website. Can you please provide the link to the actual data?

The data used in this study were provided by EyePACS, Inc, a third party provider. Data are available from EyePacs Inc. (contact@eyepacs.org) upon request for a fee. The link was provided in error and this has been corrected. We have extended the data availability statement for the fundus images. The Kermani dataset is available with the respective paper online.

Minor comments:

• Abstract:

o What does it mean to look superficially realistic if experts were able to discern real vs generated CFIs?

We think the reviewer refers to the OCT images – in this case, when simply looking at an individual example, this appeared to look like an OCT image should. Yet, when pitched against two real images in a 3-way odd-one-out task, ophthalmologists were able to pick them out with better than chance performance. We stress that this task is very sensitive and passing it as a test is a high bar – typically, ophthalmologists attributed their ability to pick out the counterfactual OCT image to too regular lesion structure. We have rephrased this part of the abstract and emphasized this point in the text.

• Methods:

o It seems that a single person could have contributed more than one image in the EyePacs dataset, are there concerns that these individuals may be biasing the data as they may be represented more than once (while others may be represented only once)?

This is true – on average about 3 images. We made sure that all images of one person were assigned to the training, validation or test set, respectively.

o Did the Benitez or FGADR need image quality checks?

We did not perform additional quality control on these datasets, since they were used only for training the diffusion model and not the classification models. Their purpose was to increase the number of DR samples to which diffusion model is exposed to during training, so these datasets were useful since they had a larger number of images with lesions. From visual inspection, we also found that while some images might be slightly noisy, there were no issues like in the Eyepacs Inc. Dataset, where a large number images before quality filter were completely dark or oversaturated, which we found impacts image generation performance dramatically.

o Sentence for “As we require a well-performing..” in section 2.5 seems to have been written incorrectly (grammar is off).

Thanks for pointing this out. We corrected it.

o What defines an AI expert?

We now call them AI researchers throughout the paper. These were members of the research group, as pointed out in Sec. 2.7, working on AI for ophthalmology but without clinical training.

• Results:

o What does “GT” stand for in figure 2? It would be useful to include the abbreviations and their corresponding meanings within the respective figure legend.

Thanks for pointing this out. We went through the legends again and explain abbreviations new to respective figure.

o There appears to be some speculation in the results that should be left for the discussion.

We agree with the reviewer that our results section already contains some sentences regarding the interpretation of our findings. We would like to keep it this way, as we think it is necessary for readability.

o Using the phrase “almost impossible to detect” is strong language.

We agree – rephrased.

o Is there a way to quantitatively back up the statement: “Upon visual inspection, we found that the diffusion counterfactuals seemed to effectively capture salient features of the various classes.”?

We removed the sentence from this paragraph as it did not really belong there. We first describe now qualitatively the images and why we think that the changes appear superficially realistic and then present the quantitative test, the expert study.

o Should the results in table 5 be corrected for the large differences in the abilities of the ophthalmologist and AI expert in being able to detect the correct image? I would also suggest to consistently call them either AI experts or researchers, and not change between the titles.

This is captured by the model factor “Ophthalmologists vs. AI researchers”. Here, an odds ratio of 0.66 and 0.44 indicates that AI researchers are significantly less likely than ophthalmologists to detect counterfactual images among real ones (for fundus and OCT, respectively). Regarding the label, we call them AI researchers throughout now.

Reviewer #3: Summary of key findings: this research introduces an AI algorithm that generates realistic counterfactuals for retinal images using diffusion models, aiming to enhance clinical training and patient education. The algorithm combines diffusion models with classifier guidance to create images showing how a retina might appear under different conditions, such as with or without diabetic retinopathy. The generated images are validated by experts, demonstrating they are clinically meaningful and often indistinguishable from real retinal images. The researchers found that while generated color fundus images were very realistic, the OCT images, while superficially realistic, were easier for experts to identify as AI-generated.

We thank the reviewer for their positive comments.

GENERAL COMMENTS
From a technical standpoint, the study appears to be sound, with a well-defined research question and an appropriate methodological approach.
The manuscript under review is well-written and presented in a clear and intelligible manner.
The structure of the paper follows a logical progression and figures are clear.
The data presented are consistent with the stated hypotheses, and the conclusions drawn are supported by the findings. Additionally, the code and data used in the research are available on GitHub, with the data sourced from public datasets containing a substantial amount of information. The test sets included over 38,000 healthy images in color fundus photography (CFP) and more than 34,000 healthy images in optical coherence tomography (OCT). This ensures that the findings are well-supported and that further research can build upon these extensive resources.
The code is well-organized and modular, making it suitable for large-scale projects. While the current level of commenting is reasonable, improving inline documentation, especially for complex functions, could enhance maintainability and accessibility for future users.

We thank the reviewer for appreciating our paper!

MAJOR REVISION COMMENTS
• The statement: “we show that we can generate realistic counterfactual generation within the context of retinal disease detection from two retina imaging modalities, CFP and OCT” may need to be revised. Given the authors' own admission that “OCT counterfactuals were relatively easier to detect,” it would be more accurate to restrict the claim to CFP. This clarification would better align the conclusions with the study’s findings.

We rephrased.

• The statement: “diffusion probabilistic models have also been investigated for the generation of retinal fundus images [16], however, these methods focus on generation of healthy retinal fundus images” may require reconsideration. There are existing studies on the generation of CFP in disease contexts, such as for Glaucoma (e.g., “Retinal Image Synthesis for Glaucoma Assessment Using DCGAN and VAE Models”). Including a dedicated section in the introduction

discussing disease-related generative approaches would strengthen the manuscript. Claims of novelty should be restricted to diseases that have not been previously addressed.

We agree with the reviewer and we now cite the mentioned paper. Disease related techniques using GANs are discussed a few lines above the quote stated by the reviewer.

• The statement: “We found that our method generated counterfactuals by introducing or removing the necessary disease-related features as per the query” should explicitly specify that the method targets only diabetic retinopathy (DR). Authors should avoid overgeneralization in similar statements and refer to DR, not disease.

We thank the reviewer for brining this up. We went through the paper carefully to avoid overgeneralization, and specifically corrected the mentioned paragraph in the introduction.

• When restricting their analysis to DR, it seems that their results should be compared with “High-fidelity diabetic retina fundus image synthesis from freestyle lesion maps” (Hou, Biomedical Optics Express 2022). This paper appears to be a direct competitor, and it may deserve mention in the introduction, and direct comparison in Figure 3. Alternatively, the discussion section should explicitly argue the advantages of the proposed method compared to this approach

We thank the reviewer for pointing us to this particular paper, which we now cite in the Introduction. In fact, the vessel structure of the generated images in Hou et al. 2022 is highly unrealistic, as the generated images show often additional thick vessels starting on the opposite site from the optic disc (their Figure 12). This would be immediately noted by our experts, further underscoring the achievements of our method.
