# Supplementary material for: Development and validation of an AI algorithm to generate realistic and meaningful counterfactuals for retinal imaging based on diffusion models
Source: PLOS Digit Health. 2025 May 15;4(5):e0000853. doi: 10.1371/journal.pdig.0000853 (PMC12080772; doi:10.1371/journal.pdig.0000853)
Supplement: Attachment — Submitted filename: AI_DVC_Review.docx [file pdig.0000853.s013.docx]

Summary: This is a really nice manuscript that presents a novel AI-based approach to generating realistic and meaningful counterfactual retinal images using diffusion models guided by disease classifiers. The authors propose a method that can illustrate hypothetical scenarios, such as how a healthy fundus image would appear if the subject had diabetic retinopathy (DR). The study validates the approach through a combination of classifier-guided diffusion models and user studies involving ophthalmologists and AI experts. The results and methods here are very useful to the field and have potential for future clinical use, yet require a few more points to be considered before publication.

Major comments:

- Could some of the diseased counterfactuals be generated by changing the blood vessel morphology (e.g., blood vessel density, tortuosity, etc.) in the image rather than just creating lesions? This could potentially explain why you might be seeing barely noticeable lesions in some of the DR DVCs, like discussed in figure 5. Additionally, if these techniques are eventually to be used to help train opthalmologists to diagnose disease, and if DR is diagnosed by means other than just lesions, then it would be good to make sure the images being generated for counterfactual purposes reflect all the ways that DR may be diagnosed. Therefore, I think it would be useful to also test the morphological vessel changes that occur to a disease/healthy generated image. This article (<https://www.nature.com/articles/s41467-024-52334-1>) seems to show that there are quite a few morphological changes that are coincident with DR.
- The authors indicate that they used publicly available datasets (available online) but the link they shared for eyepacs data only sends you to the eyepacs news announcements part of the eyepacs website. Can you please provide the link to the actual data?

Minor comments:

- Abstract:
  - What does it mean to look superficially realistic if experts were able to discern real vs generated CFIs?
- Methods:
  - It seems that a single person could have contributed more than one image in the EyePacs dataset, are there concerns that these individuals may be biasing the data as they may be represented more than once (while others may be represented only once)?
  - Did the Benitez or FGADR need image quality checks?
  - Sentence for “As we require a well-performing..” in section 2.5 seems to have been written incorrectly (grammar is off).
  - What defines an AI expert?
- Results:
  - What does “GT” stand for in figure 2? It would be useful to include the abbreviations and their corresponding meanings within the respective figure legend.
  - There appears to be some speculation in the results that should be left for the discussion.
  - Using the phrase “almost impossible to detect” is strong language.
  - Is there a way to quantitatively back up the statement: “*Upon visual inspection, we found that the diffusion counterfactuals seemed to effectively capture salient features of the various classes.”*?
  - Should the results in table 5 be corrected for the large differences in the abilities of the ophthalmologist and AI expert in being able to detect the correct image? I would also suggest to consistently call them either AI experts or researchers, and not change between the titles.
